# Spinal metastasis from malignant meningeal intracranial hemangiopericytoma: one-staged percutaneous Onyx™ embolization and resection - a technical innovation

**DOI:** 10.1186/1477-7819-11-152

**Published:** 2013-07-11

**Authors:** Nicolai El Hindy, Adrian Ringelstein, Michael Forsting, Ulrich Sure, Oliver Mueller

**Affiliations:** 1 Department of Neurosurgery, University of Essen, Essen, 45122, Germany; 2 Institute of Diagnostic and Interventional Radiology and Neuroradiology, University of Essen, Essen, 45122, Germany

**Keywords:** Malignant hemangiopericytoma, Spinal metastasis, Percutaneous embolization, Onyx™-20

## Abstract

**Background:**

We are the first to report one-staged resection of a spinal metastasis from malignant cranial hemangiopericytoma after preoperative Onyx™-20 embolization by direct percutaneous puncture.

Spinal metastases from cranial hemangiopericytoma are extremely rare. Surgical morbidity of these highly vascularized tumours results mainly from excessive blood loss. Preoperative embolization of hyper vascular tumours has been used to reduce intraoperative blood loss for a long time. To avoid complications from arterial catheter intervention, direct percutaneous puncture has been advocated as a safe and effective alternative.

**Methods:**

A 46-year-old man with a history of malignant cranial hemangiopericytoma deriving from the left frontal skull base presented with a short history of lower back pain. A magnetic resonance imaging scan revealed an intra- and extra spinal mass lesion of the thoracic spine at Th 12. Indication for tumour resection was made and the patient’s written consent was obtained. Preoperatively, arterial catheter angiography was performed to reveal the tumour’s angioarchitecture, revealing high-flow arteriovenous shunts. In order to impede the expected perioperative blood loss, tumour embolization by direct percutaneous puncture and application of Onyx™-20 was performed prior to surgery.

**Results:**

After percutaneous Onyx™-20 embolization, complete and safe resection of the lesion could be achieved. There was only minimal blood loss perioperatively. A pathohistological report confirmed malignant, anaplastic hemangiopericytoma.

**Conclusions:**

In our case Onyx™-20 embolization via direct percutaneous puncture of a highly vascularized tumour was shown to be a safe and efficient tool prior to surgery. Despite high-flow arteriovenous shunts, direct percutaneous administration of non-adhesive ethanol liquid was an efficient alternative to transarterial catheter embolization. The perioperative blood loss could be substantially diminished.

## Background

Hemangiopericytoma (HPC) is a rare mesenchymal tumour that is predominantly found in the pelvis, retro peritoneum and lower extremities, occasionally occurring in the larynx, spleen or bones of the thorax, or the meninges [1]. HPC located intracranially account for less than 1% of central nervous system (CNS) neoplasias and less than 3% of meningeal tumours [2]. Initially, its histological resemblance of meningioma caused HPC to be classified as a subtype of this tumour entity (‘angioblastic meningioma’). Later, HPC was defined as an own tumour entity, because of distinctive clinical and histopathological features [3,4]. Its strong tendency for local recurrence and formation of extra cranial metastases led to the treatment recommendation of radical tumour resection followed by local radiotherapy [5,6]. Extra cranial metastases have been well reported, yet spinal metastases are extremely rare [7]. The high vascularity of HPC can be a challenge for the surgeon. For this, preoperative embolization of the tumour may be indicated. A common approach is the endovascular embolization via the arterial route [8,9]. Nevertheless, if the rich blood supply of the tumour consists mainly of small arteries or en-passant feeders not feasible for selective catheterization, or the venous drainage comprises pathological distended vessels, direct percutaneous computed tomography (CT)-guided targeting of the lesion and applying nonadhesive ethylene-vinyl alcohol copolymer (Onyx™) may provide a safe alternative to obliterate substantial parts of the blood supply to the tumour.

To the best of our knowledge, this is the first report of an interdisciplinary treatment of a HPC by percutaneous Onyx™ embolization and microsurgical resection. We present our result of safe excision after near complete preoperative devascularisation of a spinal metastasis of malignant HPC using a percutaneous embolization technique with Onyx™.

## Methods

**Figure 1:**
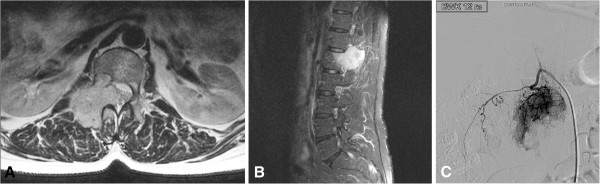
**Preoperative T2-weighted axial (A) and contrast enhanced sagittal (B) MRI revealed a mass of the L1 vertebral body, originating from the right vertebral arch, extending extra- and intraspinally with a prominent mass effect to the spinal cord.** Super selective catheter angiography of the bilateral spinal arteries Th11 to L2 revealed a major tumour blush from feeders originating from the right-sided TH12 spinal artery **(C)**.

A 46-year-old man suffering from cranial HPC initially resected in 1996 presented with a four-month history of lower back pain. A neurological examination on admission revealed local pain at the thoracolumbar junction and decreased response to pin prick and light touch at the right-sided L1 dermatome. A magnetic resonance imaging (MRI) scan of the thoracolumbar spine displayed an extensive, contrast-enhancing mass of the L1 vertebral body, originating from the right vertebral arch, extending extra- and intraspinally with a substantial mass effect to the spinal cord (Figure 1A, B). Beside the HPC resection of the left frontal skull base in 1996, initially classified HPC WHO °II, the patient’s medical charts reported no secondary malignancy. A local recurrence was re-operated upon in the year 2000, now according an anaplastic (WHO °III) HPC classification. Postoperatively, fractioned radiation therapy of 50 Gy was performed consecutively. A newly diagnosed local recurrence was treated by Gamma knife therapy in 2008. Because of local progression, another operation with gross total resection of the tumour was carried out in 2010.

Super selective angiography of the bilateral spinal arteries Th11 to L2 was performed revealing a major tumour blush with arterial supply from feeders originating from the right-sided Th12 spinal artery (Figure 1C). Endovascular embolization was not possible due to the extended diameters of the draining vessels, with the danger of uncontrolled outflow of the embolization material. Therefore, it was decided interdisciplinary not to attempt endovascular embolization, but to embolize the tumour percutaneously with nonadhesive Onyx™-20 by CT guidance before resecting the lesion within the same session.

**Figure 2:**
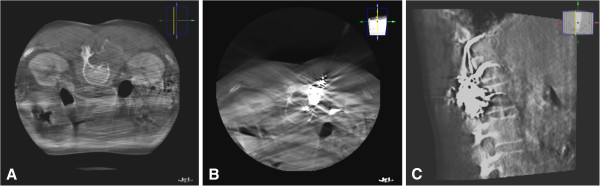
**Planning CT scan (A) with contrast enhancement revealed a hyper vascular tumour formation at the right L1 level.** Percutaneous injection of Onyx™ was performed **(B)** and intratumoural distribution was controlled by repeated CT scans **(C)**.

The procedure was performed with the patient in a prone position under general anaesthesia. A planning CT scan with contrast enhancement showed the hyper vascular tumour formation at the right L1 level (Figure 2A). Percutaneous puncture of the tumour with a 21-gauge spinal needle (Terumo, Tokyo, Japan) under sterile conditions and injection of 10 ml Onyx™-20 with changing positions of the needle was performed. Intratumoural distribution of the Onyx™-20 was controlled with repeated CT scans to exclude extravasation of embolization materials and ascertain tumour devascularisation at the same time (Figure 2B, C).

## Results and discussion

After the successful intervention the patient was transferred to the operating room (OR) and a resection of the tumour was performed under neurophysiological monitoring. Resection of the tumour was facilitated by the intratumoural Onyx™-20 cast and complete tumour removals were achieved. Perioperative blood loss was kept to a minimum (<50 ml).

**Figure 3:**
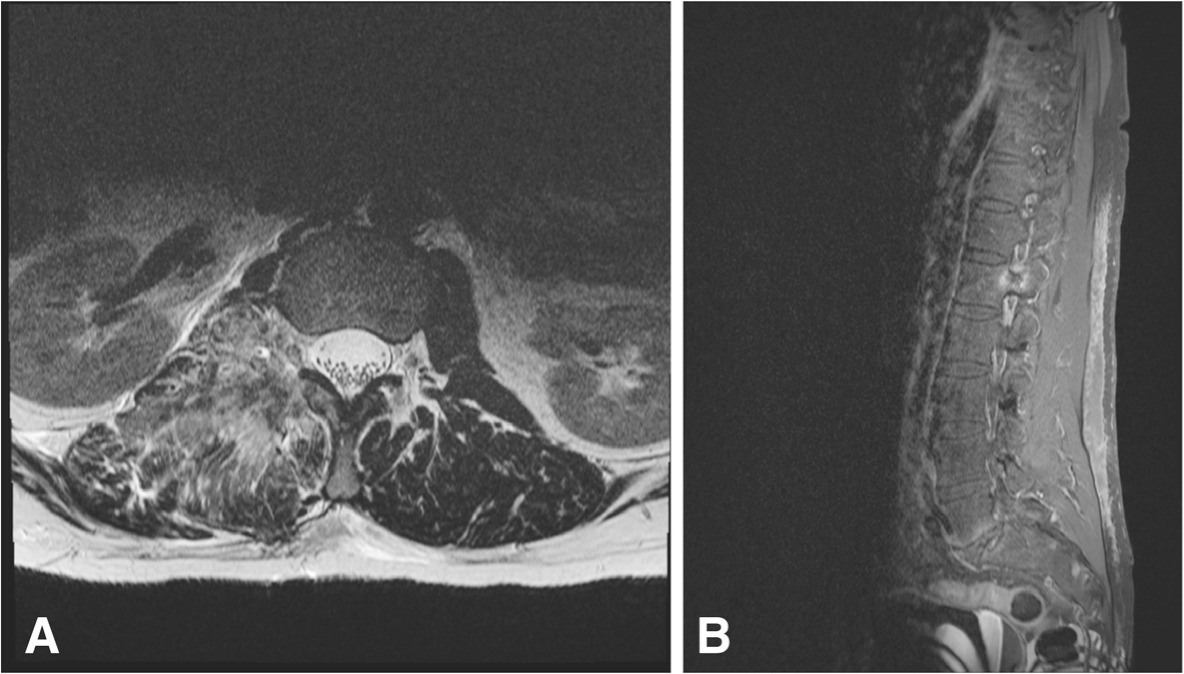
Postoperative T2-weighted, contrast-enhanced MRI confirmed complete tumour removal in axial (A) and sagittal (B) planes.

**Figure 4:**
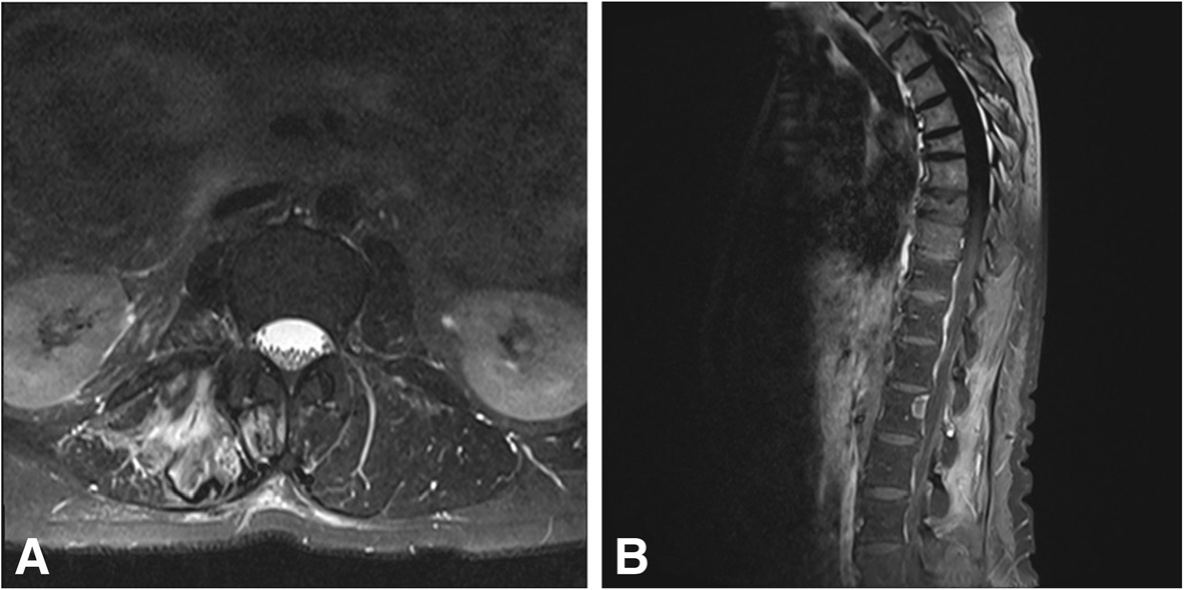
Six-month follow-up contrast-enhanced MRI confirmed complete tumour removal in axial (A) and sagittal (B) planes.

The spinal metastasis of the HPC could be resected completely and safely with an interdisciplinary approach. Histopathology workup confirmed metastasis of anaplastic (WHO °III) HPC. A postoperative MRI scan confirmed complete removal of the tumour (Figure 3A, B). Adverse events occurred neither during the interventional nor the surgical procedure, nor during the postoperative phase. The patient remained without neurological abnormalities and was discharged at day seven postoperatively. Local radiotherapy was indicated for adjunctive therapy. At the six-month follow-up, an MRI scan of the thoracolumbar spine still revealed complete removal of the tumour without evidence of recurrence (Figure 4A, B). The neurological examination was without pathological finding.

Spinal metastases of HPC are rare, yet highly vascularized, tumours. Hence, sole surgical removal without preoperatively diminishing the blood supply of the tumour might lead to a significant blood loss associated with a high peri- and postoperative morbidity and incomplete tumour resection, which in turn is associated with a lower progression-free survival period [10]. Therefore, complete resection followed by postoperative radiotherapy is considered the best treatment option for metastasized HPC and should be the goal of any surgical treatment [6]. Preoperative interventional tumour devascularisation reduces the intraoperative blood loss, additionally warranting a sharply defined dissection plane and probably facilitating gross total tumour resection. Several techniques for embolization to hypervascularized tumours, comprising an endovascular (arterial, venous, combined) or direct percutaneous route with various embolic materials have been reported in the literature [8,9,11].

Preoperative embolization of a spinal metastasis of a meningeal HPC has been only reported on a single occasion so far [9]. Direct percutaneous puncture and embolization, reported in several other highly vascularized tumours, has never been applied to HPC before [11,12].

Here, we report the preoperative embolization of a spinal metastasis of malignant cranial HPC by direct percutaneous puncture prior to surgical removal. Complete obliteration could be achieved by using nonadhesive Onyx™-20 as the sole embolic agent. This procedure was indicated as the extended and dilated vessels of the venous drainage consecutively leading to high arterio-venous shunts hindered transarterial embolization due to the risk of uncontrolled distribution of Onyx™-20 beyond the tumour bed. Moreover, tiny feeding arteries, not feasible for selective catheterization were amenable to percutaneous injections of Onyx™-20, hence leading to gradual devascularisation of the complete tumour. There are several embolic agents comprising mechanical devices, particles and fluids. According to the literature, the use of Onyx™-20 as a nonadhesive liquid embolic agent proved to be safe because the injection rate chosen can be very slow, resembling a lava-like flow [12]. Thus, the velocity of the Onyx™-20 distribution is much more controllable than that using conventional acrylates [11], reducing the risk of migration of embolic material in adjacent vital arteries and veins by retrograde filling of arterial feeders and ante grade filling of venous vessels.

## Conclusions

In the presented case, the preoperative embolization of a hyper vascular spinal metastasis of hemangiopericytoma with Onyx™-20 by percutaneous puncture was shown to be a safe and efficient technique. In cases with high AV shunt, direct puncture and embolization seems to be superior to transarterial catheter embolization, reducing the risks of uncontrollable outflow of embolic agents via the pathological angioarchitecture of the tumour. Percutaneous tumour embolization with immediate surgical intervention should be considered for large hyper vascular spinal tumours with high AV shunts as it may decrease the risk of intraoperative haemorrhage and facilitate gross total resection. However, we believe that more prospective studies have to be initiated to evaluate the possible beneficial effect of the direct percutaneous embolization of hypervascularized tumours prior to surgery.

## Consent

Written informed consent was obtained from the patient for the publication of this report and any accompanying images.

## Abbreviations

CNS: Central nervous system; CT: Computed tomography; HPC: Hemangiopericytoma; MRI: Magnetic resonance imaging.

## Competing interests

The authors declare that they have no competing interests.

## Authors’ contribution

NE, OM, and AR carried out the procedures. US and MF participated in the design and coordination of the study and helped to draft the manuscript. All authors read and approved the final manuscript.
